# The EORTC QLU-C10D is a valid cancer-specific preference-based measure for cost-utility and health technology assessment in the Netherlands

**DOI:** 10.1007/s10198-024-01670-6

**Published:** 2024-03-14

**Authors:** Micha J. Pilz, Simon Seyringer, Lára R. Hallsson, Andrew Bottomley, Femke Jansen, Madeleine T. King, Richard Norman, Marianne J. Rutten, Irma M. Verdonck-de Leeuw, Peter D. Siersema, Eva Maria Gamper

**Affiliations:** 1 University Hospital of Psychiatry II, Medical University Innsbruck, Innsbruck, Austria; 2 Institute of Public Health, Medical Decision Making and Health Technology Assessment, Department of Public Health, Health Services Research and Health Technology Assessment, UMIT TIROL - University for Health Sciences and Technology, Hall, I.T. Austria; 3 Department of Nuclear Medicine, Medical University of Innsbruck, 6020 Innsbruck, Austria; 4 Quality of Life Department, European Organisation for Research and Treatment of Cancer, Brussels, Belgium; 5 Department Otolaryngology-Head and Neck Surgery, Amsterdam UMC Location Vrije Universiteit Amsterdam, De Boelelaan 1117, Amsterdam, The Netherlands; 6 Cancer Center Amsterdam, Treatment and Quality of Life, Amsterdam, The Netherlands; 7 Department Clinical, Neuro and Developmental Psychology, Vrije Universiteit Amsterdam, Van Der Boechorststraat 7-9, Amsterdam, The Netherlands; 8 School of Psychology, University of Sydney, New South Wales, Australia; 9 School of Population Health, Curtin University, Perth, WA Australia; 10 Center of Gynaecologic Oncology Amsterdam, Amsterdam UMC, Amsterdam, The Netherlands; 11 Amsterdam Public Health, Mental Health, Amsterdam, The Netherlands; 12 Department of Gastroenterology and Hepatology, Radboud University Medical Center, Nijmegen, The Netherlands; 13 Department of Gastroenterology and Hepatology, Erasmus MC/University Medical Center, Rotterdam, The Netherlands

**Keywords:** Cancer-specific preference-based measure, EORTC QLU-C10D, EQ-5D-3L, Validity, Responsiveness, I180

## Abstract

**Background:**

Cost-utility analysis typically relies on preference-based measures (PBMs). While generic PBMs are widely used, disease-specific PBMs can capture aspects relevant for certain patient populations. Here the EORTC QLU-C10D, a cancer-specific PBM based on the QLQ-C30, is validated using Dutch trial data with the EQ-5D-3L as a generic comparator measure.

**Methods:**

We retrospectively analysed data from four Dutch randomised controlled trials (RCTs) comprising the EORTC QLQ-C30 and the EQ-5D-3L. Respective Dutch value sets were applied. Correlations between the instruments were calculated for domains and index scores. Bland–Altman plots and intra-class correlations (ICC) displayed agreement between the measures. Independent and paired *t*-tests, effect sizes and relative validity indices were used to determine the instruments’ performance in detecting clinically known-group differences and health changes over time.

**Results:**

We analysed data from 602 cancer patients from four different trials. In overall, the EORTC QLU-C10D showed good relative validity with the EQ-5D-3L as a comparator (correlations of index scores *r* = 0.53–0.75, ICCs 0.686–0.808, conceptually similar domains showed higher correlations than dissimilar domains). Most importantly, it detected 63% of expected clinical group differences and 50% of changes over time in patients undergoing treatment. Both instruments showed poor performance in survivors. Detection rate and measurement efficiency were clearly higher for the QLU-C10D than for the EQ-5D-3L.

**Conclusions:**

The Dutch EORTC QLU-C10D showed good comparative validity in patients undergoing treatment. Our results underline the benefit that can be achieved by using a cancer-specific PBM for generating health utilities for cancer patients from a measurement perspective.

## Introduction

Health systems face increasing pressure to scrutinise healthcare expenditures and allocate scarce resources in the most effective manner. Therefore, an increased utilisation of cost-utility analysis (CUA) is observed, since CUA allows policy makers to directly compare the clinical benefits and economic costs of different healthcare interventions [1]. In CUAs, quality-adjusted life years (QALYs) commonly serve as an outcome parameter combining quality of life, expressed as health utilities between 1 for perfect health and 0 for death, and lifespan into a single metric [2]. QALYs constitute the most regarded single indicator in health economics [3]. To estimate health utilities, direct preference assessments and preference-based measures (PBMs) are two commonly applied methods [4]. Direct preference assessments such as time-trade-off, standard gamble or visual analogue scales are known to be resource-intensive and time-consuming [5]. Estimating health utilities utilising PBMs provides a convenient alternative and has therefore gained increasing attention [5]. PBMs are based on a health state classification system as well as utility decrements, usually derived from the general population, to determine health state values [6, 7]. Several generic PBMs, such as the Health Utility Index (HUI) [8], the EuroQol 5-Dimensions (3 level and 5 level version-EQ-5D-3L and EQ-5D-5L) [9, 10] and the Short Form Six-Dimensions (SF-6D) [6] have been developed. Generic PBMs include general and universally applicable domains, such as physical functioning, role functioning, emotional functioning, and pain. They can, therefore, be applied in a broad range of medical conditions and make results comparable across disease groups and patient populations [11]. Currently, generic PBMs are primarily used for CUA assessments in many countries [12].

However, generic instruments have been criticised as they might fail to capture important health-related quality of life (HRQOL) symptom and functioning domains [13] such as nausea, fatigue, appetite loss in cancer patients [7]. Therefore, any impact of treatments on these domains cannot be accounted for when performing CUAs when using generic PBMs to evaluate interventions for cancer. Due to these considerations, disease specific instruments have evolved, allowing the estimation of CUA for certain patient populations [11, 14–16].

The Multi-Attribute Utility in Cancer (MAUCa) Consortium [7, 17] and the European Organisation of Research and Treatment of Cancer (EORTC) [18–21] recently developed a disease specific PBM for the cancer patient population—the EORTC Quality of Life Utility-Core 10 dimensions (EORTC QLU-C10D). For its development, the structure and content of the widely used HRQOL questionnaire EORTC QLQ-C30 was utilised to identify the most relevant HRQOL domains for cancer patients (results reported elsewhere) [7]. The EORTC QLU-C10D has been designed as a scoring algorithm for the EORTC QLQ-C30, allowing the calculation of utility values from QLQ-C30 scores [17–25].

There is a wide discussion in the literature regarding the most appropriate PBM in certain settings (e.g. palliative care, elderly people) and conditions (e.g. cancer, chronic disease) [14, 26–28]. The issue of generic versus disease specific PBMs is of particular interest, with psychometric characteristics of sensitivity (detecting health status differences) and responsiveness (detecting health status changes over time) highlighted as particularly important [26, 29]. To date, several empirical studies have investigated the measurement properties of different PBMs [28–33], with some scrutinising the sensitivity and responsiveness in various patient populations and disease categories [32, 34, 35]. In some cases, the disease-specific PBM had superior measurement attributes compared to the generic PBM [29, 32, 33, 35]. This methodological discussion is valuable as health utilities and CUAs are used by health care authorities for reimbursement decisions [36] and, therefore, determine which treatments will be reimbursed. Empirical evidence in a wide range of settings is essential to test theoretical arguments about the relative advantages of generic versus disease-specific PBMS, and the conditions under which these arguments hold or do not hold.

The aim of our study was to assess the validity of the EORTC QLU-C10D for use in the Dutch cancer patient population complying with the CONSORT quality criteria for health status questionnaires [37]. We evaluated the comparative clinical validity of the cancer specific EORTC QLU-C10D using the well-established EQ-5D-3L as the comparator measure. This includes the evaluation of floor and ceiling effects, correlations of index and domain scores, Bland–Altman plots, sensitivity for known-group differences between clinically defined groups, responsiveness (i.e. the ability to detect change in the health status), and relative efficiency (i.e. the statistical efficiency of the QLU-C10D to detect differences in health statuses in comparison to the EQ-5D-3L). We draw on data from four RCTs that investigated different healthcare interventions in the Dutch cancer patient population targeting HRQOL as secondary outcomes [38–41]. In all of them, EORTC QLQ-C30 as well as EQ-5D-3L data were collected, allowing the retrospective validation of the Dutch version of the EORTC QLU-C10D.

## Methods

### The instruments EORTC QLU-C10D

The EORTC QLU-C10D [7] is a recently developed cancer-specific PBM. It has been derived from the most widely used HRQOL questionnaire in cancer research—the EORTC QLQ-C30 [42]. Thirteen of its 30 items have been selected to form ten domains constituting the QLU-C10D health state classification system [7]. These domains are physical functioning, role functioning, social functioning, emotional functioning, pain, fatigue, sleep, appetite, nausea, and bowel problems. Each domain consists of four severity levels (“not at all”, “a little”, “moderate”, “very much”). Preference-based scoring algorithms have been developed for a range of countries [17–21], including recently the Netherlands [19]. Table 1 shows QLU-C10D domains and health state descriptions as well as conceptually similar counterpart domains of the EQ-5D-3L.

**Table 1:** QLU-C10D and EQ-5D domain and health state description analogies

Counterpart domains					
QLU-C10D			EQ-5D-3L		
Domain	Level	Health state descriptions	Domain	Level	Health state descriptions
Physical functioning	1	No trouble taking a long walk outside of the house	Mobility	1	No problems in walking about
	2	No trouble taking a short walk outside of the house, but at least a little trouble taking a long walk		2	Some problems in walking about
	3	At least a little trouble taking a short walk outside of the house, and at least a little trouble taking a long walk		3	Confined to bed
	4	Quite a bit or very much trouble taking a short walk outside the house			
Role functioning	1	Not at all limited in pursuing work or other daily activities	Usual activities (*e.g. work, study, housework, family or leisure activities)*	1	No problems with performing usual activities
	2	A little limited in pursuing work or other daily activities		2	Some problems with performing usual activities
	3	Quite a bit limited in pursuing work or other daily activities		3	Unable to perform usual activities
	4	Very much limited in pursuing work or other daily activities			
Social functioning	1	Physical condition or medical treatment interferes **not at all** with social or family life	Usual activities (*e.g. work, study, housework, family or leisure activities)*	1	No problems with performing usual activities
	2	Physical condition or medical treatment interferes **a little** with social or family life		2	Some problems with performing usual activities
	3	Physical condition or medical treatment interferes **quite a bit** with social or family life		3	Unable to perform usual activities
	4	Physical condition or medical treatment interferes **very much** with social or family life			
Emotional functioning	1	**not at all** feeling depressed	Anxiety/depression	1	Not anxious or depressed
	2	feeling **a little** depressed		2	Moderately anxious or depressed
	3	feeling **quite a bit** depressed		3	Extremely anxious or depressed
	4	feeling **very much** depressed			
Pain	1	**no** pain	Pain/discomfort	1	No pain or discomfort
	2	**a little** pain		2	Moderate pain or discomfort
	3	**quite a bit** pain		3	Extreme pain or discomfort
	4	**very much** pain			
*Domains without counterpart*					
*QLUC10D*			*EQ-5D*		
Fatigue	1	**not at all** tired	Self-Care	1	No problems with self-care
	2	**a little** pain		2	Some problems washing or dressing
	3	**quite a bit** pain		3	Unable to wash or dress
	4	**very much** pain			
Sleep disturbance	1	**no** trouble sleeping			
	2	**a little** trouble sleeping			
	3	**quite a bit** trouble sleeping			
	4	**very much** trouble sleeping			
Appetite loss	1	**not at all** lacking appetite			
	2	**a little** lacking appetite			
	3	**quite a bit** lacking appetite			
	4	**very much** lacking appetite			
Nausea	1	**not at all** feeling			
	2	nauseated **a little** feeling nauseated			
	3	**quite a bit** feeling nauseated			
	4	**very much** feeling nauseated			
Bowel problems	1	**no** constipation or diarrhoea			
	2	**a little** constipation or diarrhoea			
	3	**quite a bit** constipation or diarrhoea			
	4	**very much** constipation or diarrhoea			

### EQ-5D-3L

The EQ-5D-3L was developed in 1990 as a simple, standardised, generic HRQOL questionnaire that can also be applied as a generic PBM [43]. Its five domains are mobility, self-care, usual activities, pain/discomfort, and anxiety/depression. The EQ-5D-3L has three response levels for each domain (“no problems”, “some problems”, “severe problems/unable to”). Value sets are available for a range of countries [44–47]; the Dutch value sets were published in 2006 [44]. Table 1 shows EQ-5D-3L domains and health state descriptions as well as theoretical counterpart domains of the QLU-C10D.

### Data description

In this retrospective analysis, data from four multicentre RCTs were used, which assessed HRQOL in a broad range of cancer patients and interventions in the Netherlands. To be eligible, patients had to have completed the EORTC QLQ-C30 and the EQ-5D-3L at the same measurement time points. The first RCT is the SIREC study (*n* = 209; 199 eligible for our analysis), which was conducted in nine Dutch hospitals and investigated the effect of stent insertion vs. brachytherapy on dysphagia relief in patients with oesophagus cancer [39]. The second RCT examined the effect of a Stepped Care (SC) (*n* = 156; 147 patients eligible for our analysis) approach of psychosocial interventions versus care-as-usual on psychological distress and HRQOL in head and neck cancer and lung cancer patients who were not in active anti-tumor treatment [40]. The third RCT investigated the effect of meaning-centred group psychotherapy (MCGP-CS) vs. supportive group psychotherapy (SGP) vs. care-as-usual on personal meaning and HRQOL in (*n* = 170; 168 cancer survivors eligible for our analysis) Dutch cancer survivors [41]. The fourth RCT was conducted as a multicentre study in eight Dutch gynaecological centres, investigating the effects of diagnostic laparoscopy on treatment decision making, such as applying primary cytoreductive surgery (LapOvCa), in 201 patients with advanced ovarian cancer (78 patients eligible for our analysis) [38]. All trials assessed the EORTC QLQ-C30 and the EQ-5D-3L as secondary endpoints. Patient data were included in the current analysis if EORTC QLU-C10D and EQ-5D-3L longitudinal data was available. Details regarding the study design and sampling procedures of the RCTs have been reported previously [38–41].

### Statistical analysis

Data from all four RCTs were analysed separately as pooling of data was not appropriate due to the differing study designs. Sample characteristics were presented as absolute and relative frequencies, means, and standard deviations. Utility scoring was conducted using the Dutch value sets for the EORTC QLU-C10D and the EQ-5D-3L [19, 44].

To investigate the clinical validity, ceiling and floor effects for the PBMs were calculated as the percentage reaching maximum/minimum health utility scores for each instrument. According to Terwee et al. [37], substantial floor or ceiling effects are present if more than 15% report the lowest or highest score possible, respectively. Furthermore, Spearman correlation coefficients were calculated to evaluate the construct and content validity by correlating the utility value index scores and domain pairs considering coefficients of 0.1–0.3 as weak, 0.4–0.6 as moderate, and 0.7–0.9 as strong [48]. On the domain level, some cancer-specific aspects from the QLU-C10D conceptually differ from the generic EQ-5D domains (displayed in Table 1) and were therefore expected to show low correlations (divergent validity). For domains with conceptually similar content (EQ-5D mobility and QLU-C10D physical functioning, EQ-5D usual activities and QLU-C10D role- and social functioning, EQ-5D pain/discomfort and QLU-C10D pain, EQ-5D anxiety/depression and QLU-C10D emotional functioning), moderate to strong correlations were expected (convergent validity). Strong correlations were expected between the index scores of the two PBMs. As measures of agreement between the QLU-C10D and the comparator measure, intra-class correlations (ICCs) of index scores were calculated. To further scrutinize the agreement between the scores across the measurement ranges, Bland–Altman-Plots were created. These were used to display systematic (dis)agreements between the measures.

Sensitivity and responsiveness were investigated by scrutinising the PBMs ability to discriminate between clinical known-groups and between time points for which health states were expected to differ, respectively. Sensitivity was investigated in cross-sectional groups at baseline (for the “dysphagia improved” and “retreatment” groups in the SIREC trial and all groups in the LapOvCa trial, follow-up data was used). The definition of clinical known-groups was performed according to what was known from the literature, complemented by the expert opinion of co-author clinicians. That is, we considered what could be expected with regard to health or HRQOL differences based on the results from the respective included trials [38–41], groups based on WHO performance status [49], type of cancer treatment [50–52], working status [53], histology [54], or tumour stage [55]. Selection of variables for known-group comparisons and the expected direction of utility differences are reported in Table 2.

**Table 2:** Sociodemographic data and treatment information across four RCTs, including variables used for known group comparisons*

	SIREC (*N* = 209)	SC (*N* = 147)	MCGP-CS (*N* = 168)	LapOvCa (*N* = 78)**
**Mean age (SD)**	68.7 (11.6)	62.8 (9.3)	57.1 (10.2)	62.4 (9.3)
**Sex**				
Male	162 (77.5%)	83 (58.9%)	29 (17.3%)	0 (0.0%)
Female	47 (22.5%)	58 (41.1%)	139 (82.7%)	78 (100.0%)
**Living arrangements**				
Married/living together	-	96 (68.1%)	133 (79.1%)	-
Unmarried/divorced/widowed	-	45 (31.9%)	33 (19.6%)	-
Other	-	-	4 (2.3%)	-
**Paid work**				
No	-	98 (69.5%)	77 (45.8%)	-
Yes	-	43 (30.5%)	88 (52.4%)	-
Missing	-	-	3 (1.8%)	-
Variables for known group comparisons*	**Treatment** stent < brachytherapy **WHO-PS** 0–2 > 3–4 **Histology** Squamous cell vs. Adeno Cell **Previous CTX** no > yes **Improved dysphagia at day 30** yes > no **Persistent dysphagia at day 30** yes < no **Retreatment after 30 days** yes > no	**Treatment** Stepped Care > Care as usual **Paid work (working age < 66yrs)** yes > no **Time since treatment** less than 7 months < more than 7 months **Treatment** Unimodal > Multimodal **Tumour stage** local > advanced	**Treatment** Meaning-Centred Group Psychotherapy ~ Supportive Group Psychotherapy ~ Care as usual **Primary tumour** breast ~ colon **Treatment** chemoradiation ~ radiotherapy **Hormonal therapy received** yes < no	**Futile Laparotomy** no > yes **Formal care** no > yes **Informal care** no > yes **Figo stage** < IIIc > IIIc/IV **WHO PS** 1 > 2–4 **Histology** malign < benign **Residual disease**; none > + 1 cm
**Tumour location**				
Oesophag	179 (85.6%)	–	–	–
Oesophag. Junct	30 (14.4%)	–	–	–
Lip/oral cavity/oropharynx	–	69 (48.9%)	–	–
Hypopharynx/larynx	–	36 (26.2%)	–	–
Other head and neck cancers	–	27 (18.4%)	–	–
Lung	–	9 (6.4%)	–	–
Breast	–	–	112 (66.7%)	–
Colon	–	–	36 (21.4%)	–
Ovaria	–	–	-	78 (100%)
Other			20 (11.9%)	
**Tumour treatment**				
Surgery	–	33 (23.4%)	167 (99.4%)	60 (76.9%)
Radiotherapy	–	34 (24.1%)	95 (56.5%)	–
Chemotherapy	179 (85.6%)	24 (17.0%)	95 (56.5%)	8 (10.3%)
Hormonal therapy	–	–	80 (47.6%)	–
Surgery and radiotherapy	–	38 (27.0%)	–	–
Surgery and chemoradiation	–	8 (5.7%)	–	–
Surgery and chemotherapy	–	4 (2.8%)-	–	–
Brachytherapy	101 (48.3%)	–	–	–
Stent	108 (51.7%)		–	–
**WHO performance status at baseline (t0)**				
0—Fully active	79 (37.8%)	–	–	–
1—Restricted in physically strenuous activity	66 (31.6%)	–	–	45 (57.69%)
2—unable to carry out any work activities	39 (18.7%)	–	–	32 (41.03%)
3—confined to bed or chair more than 50% of waking hours	19 (9.1%)	–	–	1 (1.28%)
4—completely disabled	3 (1.4%)	–	–	–
Missing	3 (1.4%)	–	–	–
**Tumor stage**				
UICC I–II	–	60 (42.6%)	–	–
UICC III–IV	–	72 (51.1%)	–	–
Missing	–	9 (6.4%)	–	–
**FIGO stage**				
FIGO I–IIIb	–	–	–	25 (32.1%)
FIGO IIIc—IV	–	–	–	53 (68.0%)
**Mean Months since last treatment**				–
< 7 months	–	48 (34.0%)	–	
7–12 months	–	24 (17.0%)	–	–
> 12 months	–	69 (48.9%)	–	–

^*^Variables for known-group comparisons were selected based on availability in the trial data sets; the direction of expected differences is indicated by < (utilities of group 1 is expected to be smaller than utilities of group 2), > (utilities of group 1 is expected to be greater than utilities of group 2), or ~ ((utilities of group 1 and 2 are expected to be similar)

^**^As the LapOvCa trial had a high missing HRQOL data rate, potential systematic bias was assessed with independent t-tests of clinical variables between included (*n* = 78) versus excluded patients (*n* = 123); there were no statistically significant differences (p-values ranged from 0.082 to 0.909)

Abbreviations: *SIREC* stent or Intraluminal Radiotherapy for inoperable Esophageal Cancer, *SC* stepped care, *MCGP-CS* Meaning Centred Group Psychotherapy for Cancer Survivors, *LapOvCa* laparoscopy to predict the result of primary cytoreductive surgery in advanced ovarian cancer patients, *UICC* Union for International Cancer Control, *FIGO* federation Internationale de Gynecolgie et d'Obstetrique, *SD* standard deviation

Differences of index scores between groups (sensitivity) and over time (responsiveness) were statistically tested using *T*-tests (independent t-tests for sensitivity and repeated measures t-tests for responsiveness). Minimal important differences for the EQ-5D-3L [56] were used to contextualise the magnitude of differences and change. Relative validity (comparative validity) indices were calculated using a three-fold approach; by calculating effect sizes, the Relative Efficiency, and the Responsiveness Index. Cohen’s D and standardised response mean (SRM) were used as effect size measures; Cohen’s D was used to estimate effect sizes between groups (sensitivity) while the SRM was used as effect size measure for change over time (responsiveness). Cohen’s D were classified as small (0.2–0.49), moderate (0.5–0.79) and large (≥ 0.8) [57]. We used the same thresholds for SRM. The Responsiveness Index (RI) was calculated for each instrument by dividing the change over time by the SD of the control condition following the methodology applied by King et al. [58]. The RI was originally developed to test an instruments’ ability to assess change between a treatment and a (stable) intervention arm [59]. When testing the RI also for further clinical variables, we additionally calculated the RI for other clinical subgroups which deviated over time (e.g., improved/persistent dysphagia, (non-) residual disease, recipient of formal care yes/no, etc.). In addition, when both instruments detected statistically significant effects, the instruments’ relative efficiency (RE) was assessed as the ratio of squared *t* values $\frac{{\left(t-\mathrm{statistic EORTC QLU}-{\text{C}}10{\text{D}}\right)}^{2}}{{\left(t-\mathrm{statistic EQ}-5{\text{D}}-3{\text{L}}\right)}^{2}}$, whereby a RE > 1 indicates a higher efficiency for the QLU-C10D and RE < 1 a higher efficiency for the EQ-5D, respectively [58, 60]. To further investigate the relative validity of responsiveness for the instruments, the difference of RI was calculated by subtracting resulting RI indexes. A positive RI difference indicates better responsiveness of the EORTC QLU-C10D, while a negative RI difference indicates superior responsiveness for the EQ-5D-3L.

To adjust for multiple testing and to keep the alpha error ≤ 5%, Bonferroni correction was applied (p-values corrected according to the number of tests performed per data set). All statistical analyses were performed using IBM SPSS 21 [61].

## Results

### Sample characteristics

The included patient data across the four RCTs represented a diverse sample with respect to sociodemographic and clinical characteristics. The mean age across the analysed studies ranged from 57.1 years for the MCGP-CS trial to 68.7 years for the SIREC trial. The proportion of male participants per study ranged from 0% for the LapOvCa trial up to 77.5% for the SIREC trial. Patients had mostly been treated with chemotherapy, surgery or radiotherapy. Cancer sites differed across the trials (SIREC: oesophagus, LapOvCa: ovaries, SC: head and neck, and lung, MCGP-CS: heterogeneous sample, including patients with breast and colon cancer). Patients were in active anti-tumour treatment in two trials (SIREC, LapOvCa) and were survivors or at post treatment (majority > 7 months) in the other two (MCGP-CS and SC). Further details are provided in Table 2.

### Ceiling and floor effects

Ceiling effects were below the 15% threshold for the EORTC QLU-C10D (0.7% in SC to 7.7% in LapOvCa) and exceeded the threshold of 15% for the EQ-5D-3L in two trials (20.8% in MCGP-CS and 15.4% in LapOvCa). Floor effects for both instruments were marginal throughout (≤ 1.3%). For details, see Table 3.

**Table 3:** Ceiling and Floor Effect for the EQ-5D-3L and the QLU-C10D

	EQ-5D-3L		QLU-C10D	
	Ceiling effect	Floor effect	Ceiling effect	Floor effect
SIREC	12.3%	0.0%	4.3%	0.0%
Stepped Care	8.5%	0.0%	0.7%	0.0%
MCGP—CS	20.8%	0.0%	4.8%	0.0%
LapOvCa	15.4%	1.3%	7.7%	1.3%

### Correlation between measures – convergent and divergent validity

Overall, Spearman’s correlation coefficient between the EQ-5D-3L and the QLU-C10D index scores were moderate to strong ranging from 0.534 to 0.749. Conceptually, similar domains showed mostly moderate correlations. For the pairs Physical Functioning—Mobility, Role Functioning—Usual Activities, and Social Functioning—Usual Activities the statistically significant correlation coefficients ranged from 0.432 to 0.711. For the domain pair Pain—Pain/Discomfort, the statistically significant correlations ranged from 0.335 to 0.697. The Emotional Functioning—Anxiety/Depression domain pair showed statistically significant moderate correlation only in the SC trial (0.352, *p* ≤ 0.001); in the other three trials, correlations were weak (0.119–0.178). Conceptually dissimilar domains mostly showed weak correlations. Only for the domain pair Physical Functioning—Usual Activities, a statistically significant correlation coefficient exceeded 0.5 points in the SIREC and LapOvCa trial, which is classified as moderate correlation. Further details are provided in Table 4.

**Table 4:** Spearman Correlations of index values and utilities per domain

	SIREC					SC					MCGP-CS					LapOvCa				
QLU-C10D	EQ5D-3L					EQ5D-3L					EQ5D-3L					EQ5D-3L				
	Index					Index					Index					Index				
Index	0.640**					0.657**					0.534**					0.749**				
	Domains					Domains					Domains					Domains				
Domains	MO	SC	PD	AD	UA	MO	SC	PD	AD	UA	MO	SC	PD	AD	UA	MO	SC	PD	AD	UA
PF	0.623*	0.441*	0.068	0.139	0.558*	0.590*	0.348*	0.291*	0.192	0.341*	0.574*	0.137	0.370*	0.248*	0.430*	0.504*	0.352*	0.320*	0.007	0.526*
RF	0.456*	0.473*	0.292*	0.272*	0.688*	0.417*	0.266*	0.332*	0.288*	0.483*	0.286*	0.227*	0.324*	0.170	0.532*	0.205	**0.270**	0.224	0.033	0.711*
SF	0.325*	0.403*	0.318*	0.228*	0.495*	0.321*	0.234*	0.372*	0.269*	0.524*	0.304*	0.114	0.079	0.267*	0.432*	0.144	0.235	0.384*	0.225	0.568*
EF	0.127	0.298*	0.120	**0.178**	0.248*	0.085	0.048	0.194	0.352*	0.222*	-0.063	-0.021	-0.016	**0.162**	0.015	0.162	-0.03	0.085	**0.119**	0.047
PA	0.079	0.095	0.501*	0.274*	0.235*	0.220*	0.170	0.414*	0.182	0.129	0.445*	0.077	0.335*	0.174	0.246*	0.372*	-0.038	0.459*	0.126	0.305*
FA	0.308*	0.316*	0.292*	0.235*	0.483*	0.214	0.061	0.365*	0.373*	0.393*	0.146	0.112	0.245*	0.175	0.299*	0.318*	0.350*	0.290	0.165	0.531*
SL	0.051	0.120	0.072	0.223*	0.217*	0.196	0.019	0.174	0.099	0.051	0.104	-0.004	0.139	0.121	0.075	0.082	-0.037	0.230	0.197	0.170
AP	0.068	0.141	0.263*	0.235*	0.164	0.154	0.154	0.315*	0.277*	0.305*	0.076	0.081	0.063	0.114	0.095	0.225	0.189	0.393*	0.344*	0.347*
NA	0.108	0.117	0.167	0.114	0.260*	0.049	0.083	0.304*	0.152	0.232*	0.217*	0.086	0.287*	0.055	0.224*	0.123	0.116	0.198	0.053	0.125
BO	0.228*	0.206*	0.162	0.117	0.193*	0.247*	-0.056	0.210	0.172	0.173	0.226*	-0.009	0.154	0.074	0.227*	0.221	0.072	0.21	0.240	0.040

Correlation coefficients of theoretically corresponding domains are formatted **bold**

*EQ-5D: MO* mobility, *SC* self care, *PD* pain and discomfort, *AD* anxiety and depression, *UA* usual activities, *QLU-C10D: PF* physical functioning, *RF* role functioning, *SF* social functioning, *EF* emotional functioning, *PA* pain, *FA* fatigue, *SL* sleep, *AP* appetite, *NA* nausea, *BO* bowel problems

^*^significant after Bonferroni correction at p < 0.05

### Agreement between measures

**Fig. 1:**
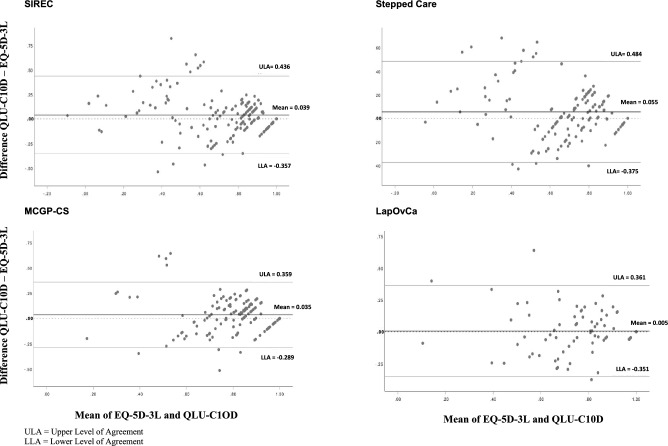
Bland–Altman Plots displaying the agreement between the EORTC QLU-C10D and the EQ-5D-3L index scores

The ICCs between the index scores of the QLU-C10D and the EQ-5D ranged from 0.686 up to 0.808. According to Cicchetti [62], these ICCs can be classified as good to excellent (see Table 5). Bland–Altman plots (Fig. 1) indicated that the QLU-C10D produced consistently higher utility values compared to the EQ-5D-3L. This systematic measurement difference ranged from 0.005 for the LapOvCa trial to 0.055 for the SC trial. In addition, the Bland–Altman-plots showed some systematic bias with score differences becoming smaller towards the upper end of the measurement scale. The distribution and span of the scores differences are presented as the level of agreements (LOA), whereby it is defined that 95% of the observed score difference lies within the LOA. The LOA ranged from 0.648 for the MCGP-CS trial up to 0.859 for the SIREC trial (see Fig. 1).

**Table 5:** Intra-class correlation from the EQ-5D-3L and the EORTC QLU-C10D across four RCTs

	ICC^a^	CI^b^	*p* value
SIREC	0.808	0.745–0.856	< 0.001
SC	0.740	0.633–0.815	< 0.001
MCGP-CS	0.686	0.573–0.769	< 0.001
LapOvCa	0.751	0.609–0.842	< 0.001

^a^Intra-class correlation

^b^CI: 95% Confidence Interval

### Sensitivity and responsiveness analysis

#### Sensitivity

**Fig. 2:**
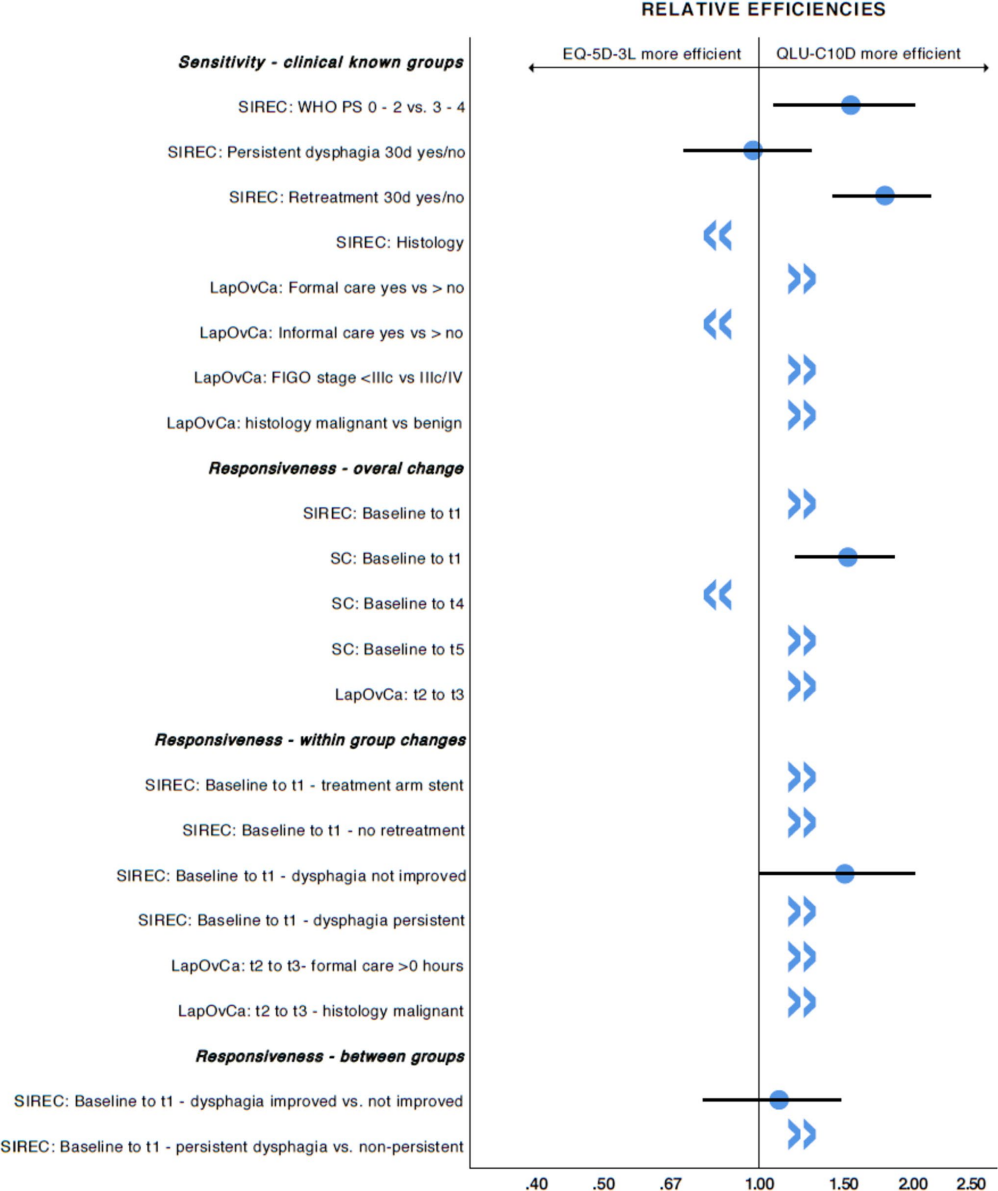
Relative Efficiencies and detection of clinical group differences/change over time. >> The QLU-C10D detected a statistically significant difference where the EQ-5D-3L did not. << The EQ-5D-3L detected a statistically significant difference where the QLU-C10D did not. *Blue filled circle* represent relative efficiency with confidence interval (only calculated if both instruments found statistically significant differences/changes)

The results of the known-group comparisons can be seen in detail in Table S1. The EORTC QLU-C10D and the EQ-5D-3L both detected statistically significant differences of index scores in the same three groups (WHO performance status 0–2 vs. 3–4, persistent dysphagia at day 30 yes vs. no, retreatment needed yes vs. no) with p-values ≤ 0.05 and REs twice in favour of the QLU-C10D (see Fig. 2). The QLU-C10D detected three additional differences not detected by the EQ-5D-3L (histology malignant vs. benign in ovarian cancer, formal care needed yes vs. no, FIGO stage < IIIc vs IIIc/IV) and the EQ-5D-3L found two additional difference that the QLU-C10D did not detect (histology in oesophagus cancer, informal care needed yes vs. no). All significant utility score differences were in the expected directions (e.g., patients with higher WHO-PS had lower utility values; patients with higher FIGO staging had lower utility values; patients with benign histology had higher utility values). The results indicate a good sensitivity for the EORTC QLU-C10D to detect differences in health states. When compared to the EQ-5D-3L the EORTC QLU-C10D detected these cross-sectional health state differences with a higher efficiency in 5 from 8 (62.5%) comparisons (Fig. 2).

#### Responsiveness

The EORTC QLU-C10D detected four statistically significant changes of index scores for overall changes over time (SC: t0 to t1, *p* < 0.013), three of which were not detected by the EQ-5D-3L (SIREC: t0 to t1; *p* < 0.001, SC: t0 to t5, LapOvCa: t2 to t3) (Table S2). Where both instruments measured differences with statistical significance (SC: t0 to t1), the QLU-C10D had a higher relative efficiency in detecting this difference. (Table S2 and Fig. 2). All test for overall change showed a score change in the expected direction, e.g. lower follow-up scores for the SIREC where health states were expected to deteriorate over time, and higher follow-up utility values for the LapOvCa trial (after treatment completion) and the SC trial where health states were expected to improve over time, indicating good responsiveness for the QLU-C10D.

Analyses of changes over time within (treatment) groups (see Table S3) showed that the EORTC QLU-C10D detected six groups with statistically significant change, while the EQ-5D-3L was able to identify one groups with statistically significant changes over time. Most of these statistically significant change scores were congruent with the hypothesised direction, e.g., utility scores deteriorated for patients with stent treatment, or with non-improved and persistent dysphagia in the SIREC trial, and improvement for malignant patients and patients relying on formal care after treatment completion in the LapOvCa trial. Additionally, the SRM was calculated for (treatment) group comparisons. There was no group comparison with a high SRM, four SRMs of the EORTC QLU-C10D and one SRM of the EQ-5D-3L were classified as moderate, respectively. In the comparison where both instruments detected statistically significant changes, the EORTC QLU-C10D showed a higher RE compared to the EQ-5D-3L (see Fig. 2).

For the responsiveness analysis for change over time between (treatment) groups, we performed 14 between-group analyses. For these 14 comparisons, the EORTC QLU-C10D was able to identify two statistically significant differences between groups—change over time differences, while the EQ-5D-3L identified one statistically significant difference for change over time between (treatment) groups. Again, the observed statistically significant differences for change over time between groups are in line with the expected direction, showing higher utility values for patients with improved/non-persistent dysphagia in the SIREC-trial. Where applicable, RE was in favour of the EORTC QLU-C10D (see Fig. 2). As an additional parameter of responsiveness, the RI showed that the EORTC QLU-C10D showed a higher responsiveness (sensitivity to change) in eight of these comparisons, indicating a higher measurement precision for health state changes over time for the EORTC QLU-C10D. For further details, please see table S3 and Fig. 2.

## Discussion

The EORTC QLU-C10D is a cancer-specific utility instrument developed as a scoring algorithm for the widely used cancer-specific quality of life questionnaire EORTC QLQ-C30. It aims to support health economic evaluations in cancer patient populations [7]. The instrument is now in the final clinical validation process, to which this and other studies [31–33, 63] contribute. The EQ-5D-3L is a well-established and widely used PBM, making it a useful standard comparator against which to assess the clinical validity of the EORTC QLU-C10D. As our analyses have shown, the QLU-C10D measures five generic constructs similar to those included in the EQ-5D, and an additional five domains related to symptoms commonly experienced by cancer patients. Additional to the content differences, the preference weights (i.e. utility decrements), also contribute to score differences of the two PBMs. Below we discuss the various aspects of validity we assessed in this study and interpret these in the context of these two key differences [31, 32, 63] between the QLU-C10D and the EQ-5D.

The QLU-C10D’s criterion validity relative to the EQ-5D-3L is supported by adequate correlations between the QLU-C10D’s and the EQ-5D’s index scores and conceptually similar domain pairs, although correlations between counterpart domains were overall somewhat weaker than expected. There was a striking difference between expected and actual correlations for the Emotional Functioning domain of the QLU-C10D (which assesses feeling depressed) and the Anxiety/Depression domain of the EQ-5D-3L (which assesses feeling anxious or depressed). However, data from an international observational study in patients with myelodysplastic syndrome indicated that these two scales also behaved differently using the Italian tariffs [32]. As hypothesised in that paper, this may be because the EQ-5D domain includes anxiety which very well can be present without feelings of depression, while the QLU-C10D domain captures only depression, not anxiety. Aside from differences in the way the instruments ask about emotional burden, the impact of emotional functioning in the Dutch EORTC QLU-C10D value set is smaller than for other countries [17–25], and is clearly lower than the Dutch EQ-5D-3L anxiety/depression utility decrement [44]. Hence, the rather low correlation for the emotional domain may also be influenced by our use of the Dutch QLU-C10D value set where only the highest response category (highest emotional burden) has a utility decrement. It is unclear whether this expresses an informative preference or is the result of a translation issue of the word ‘depression’ in the QLQ-C30; if the latter, then the impact of emotional functioning on Dutch QLU-C10D utility may be underestimated using the current Dutch versions of the QLQ-C30 and QLU-C10D value set.

The moderate correlation of the theoretically distant domains pair Mobility and Usual Activities in the SIREC trial and LapOvCa trial might be explained by the palliative setting in the SIREC trial [64] and the advanced disease stages in the LapOvCa trial [38].

The ceiling effects for the EORTC QLU-C10D (0.7–7.7%) were clearly lower than for the EQ-5D-3L (8.5–20.8%) across the four trials. BA plots indicated that the EORTC QLU-C10D produced systemically higher utility values than the EQ-5D-3L in all four studies. The level of agreements showed a maximal range from − 0.375 to 0.484 in the SC trial, exceeding the range of any observed SD of utility values in that trial. Still, the mean difference of the scores does not exceed the minimal important difference of the EQ-5D-3L in Norwegian glioma patients [56], which we used as a crude measure to evaluate the magnitude of utility score differences. However, the BA plots also showed at least some systematic bias in all four studies, indicating that the differences between QLU-C10D scores and EQ-5D3L scores vary across the utility measurement scale. The difference in measurement precision towards the upper end of the scales (existing ceiling effects for the EQ-5D-3L) potentially contributes to the systematic difference of utility scores derived from the two instruments. Therefore, utility scores between the instruments are not interchangeable.

Most importantly for clinical validity, the QLU-C10D’s sensitivity and responsiveness were assessed. We found statistically significant differences/changes in the hypothesised direction, indicating a good construct validity and sensitivity to expected differences/changes of utility scores. There was an agreement between the QLU-C10D and EQ-5D-3L regarding the detection of expected differences. In overall, the QLU-C10D had an advantage with measurement efficiency which showed by either being the only instrument picking up a hypothesised effect or being more efficient in direct comparison with the EQ-5D-3L. This was especially true for responsiveness analyses. Higher efficiency would translate to smaller required sample sizes of future clinical studies assessing utilities. Similar results were obtained when analysing the responsiveness within and between clinical subgroups across different assessment time points, whereby the EORTC QLU-C10D showed continuously higher responsiveness within and between group comparisons over time. Shaw et al. [33] reported similar findings of improved sensitivity and responsiveness for the UK version of the EORTC QLU-C10D in patients receiving Nivolumab. Additionally, Bulamu et al. reported a superior responsiveness of the EORTC QLU-C10D over the EQ-5D-3L in patients undergoing esophagectomy.

The ability to discriminate was limited in some hypothesised known-group and responsiveness analyses. In a range of analyses neither of the PBM instruments detected a statistically significant difference/change. The included RCTs had HRQOL as secondary outcomes and therefore might be underpowered for HRQOL analyses. It can be surmised that some known-group differences went undetected. A number of effects were found despite underpowered subgroup analyses and, therefore, allow drawing solid conclusions on the applicability of the Dutch QLU-C10D as these would not disappear with higher power.

Variations in psychometric properties across diverse populations, disease- and treatment groups are no surprise [65] and it is important to know the instruments characteristics for a specific target population. In our analyses, we attribute the differences in psychometric performances to the distinct clinical contexts (e.g. inpatients vs. outpatients), the trial population (actively treated cancer patients vs. cancer survivors; different cancer sites), and the interventions (medical vs. psychosocial). For example, in the MCGP-CS trial, both PBMs showed poor performance. As in the original MCGP-CS publications [41, 66], there was no statistically significant difference/change reported for EORTC QLQ-C30 and EQ-5D-3L data; our findings align with this. This suggests that in cancer survivors both instruments need to be used with caution until their fitness for this purpose is explicitly evaluated. An additional possible explanation for different findings across trials might be the nature of intervention across the trials (psycho-social interventions for the MCGP-CS and SC trial vs. somatic interventions for the SIREC and LapOvCa trial) and the conceptual scale design of the PBMs. Although the psychological aspects of health (Anxiety/Depression) is one of the five domains from the EQ-5D-3L, a study has shown that the EQ-5D-3L does not sufficiently portray the influence of mental and social health [67]. Similarly, the Emotional Functioning domain, asking for depressive mood and constituting one of the ten EORTC QLU-C10D domains, was shown to have a low impact on health utility values in Dutch cancer survivors [68]. Therefore, it can be argued that the instruments’ abilities to capture the effects of emotional aspects on health utility values might be limited to some extent which could be reflected in lower sensitivity in samples where these are predominant problems. The topic of generic and disease-specific PBMs has been discussed for several years among health economists [11, 14–16, 69]. While a multitude of disease-specific instruments were developed, the National Institute for Health and Care Excellence suggests using the EQ-5D-3L as the preferred measure of health-related quality of life [70]. In the Netherlands, the use of the EQ-5D-5L is recommended, although additional PBMs may be justified to supplement evidence from the EQ-5D [71]. However, this generic approach in health economic evaluations may be reconsidered when targeting specific patient populations [27]. The EQ-5D-3L has been shown to produce valid results in a cancer patient population, yet specific conditions were identified in which it appears to be limited, and in which the EORTC QLQ-C30 seemed more adequate [15]. In addition, it is an extra questionnaire which should be filled out by the patient. The good construct and content validity of the EORTC QLU-C10D [32] in combination with its backwards compatibility with the EORTC QLQ-C30 are both favourable prerequisites for the EORTC QLU-C10D to become adopted in health economic evaluation schemes. This study contributes to this discussion on using generic or disease-specific instruments and suggests this topic needs to be explored further. Selecting the appropriate instrument is important as health utilities and the economic evaluations are increasingly used by health care authorities for decision which treatments to reimburse [36].

## Limitations

A limitation of this study is the relatively small sample size for some of the known-group comparisons. This limited statistical power such that some real differences may have gone undetected. Another limitation of this analysis is its retrospective nature; therefore, we were not able to a priori define known-group comparisons and had to rely on variables available in the data sets from the primary studies. Nonetheless, the heterogeneous nature of the interventions and clinical contexts allowed us to perform known-group analysis across patient groups with various characteristics. This validation study of the EORTC QLU-C10D relied on comparison with data obtained using the EQ-5D-3L. Using the newer version of the EuroQol measurement system, the EQ-5D-5L [10], as comparator in the Dutch cancer patient population was not possible due to the retrospective design of this study. The two additional levels of the newer version of the EQ-5D have improved its precision relative to the EQ-5D-3L [72]. However, it is not only the number of response category that affects the granularity of the measurements but also the content, and the additional disease-specific domains of the QLU-C10D are relevant here. Bulamu et al. [73] and Pan et al. [63] have compared aspects of validity and the RE of the EORTC QLU-C10D versus the EQ-5D-5L in gastric, and oesophagus cancer patients, whereby a superior relative validity was reported for the EQ-5D-5L in comparison to the findings of the 3-level version in this manuscript [63]. Further comparisons of the QLU-C10D and versus the EQ-5D-5L in other patient groups and clinical contexts are warranted to assess the generalisability of these findings. A final limitation was that we were not able to calculate and compare QALYs from the QLU-C10D and the EQ-5D as we did not have access to existing cost-effectiveness models for the four RCTs in this study. This had been done four times before [33, 74–76], exclusively showing lower incremental QALYs for the EORTC QLU-C10D compared to the EuroQOL measurement system. Where possible, future comparisons of the QLU-C10D with a generic PBM should include comparison of QALYs yielded, as this is of great relevance when choosing PBMs to conduct CUA.

## Conclusion

Using data of four Dutch RCTs in this retrospective analysis showed good psychometric properties and clinical validity of the EORTC QLU-C10D compared to the EQ-5D-3L in the Dutch cancer patient population. Our findings show promising results for the further use of the EORTC QLU-C10D when facilitating CUA for cancer patients in the Netherlands. Importantly, in the Netherlands, health technology assessments have been applied since the early 1980s, mostly for drug reimbursement decisions [71]. Additionally, it is mentioned that in the area of specialist medical care, the nature of complexity and availability of quality data poses a limitation to health economic evaluations in the Dutch context [77, 78]. It, therefore, appears that the existence of huge data registries such as PROFILES [68] containing EORTC QLQ-C30 data and the backward compatibility of the EORTC QLU-C10D with its parent instrument, bear great opportunities for maturing health economic evaluations in the Netherlands.

## Data Availability

Data may be available upon reasonable request from the corresponding author.
